# Adipose-Specific PPARα Knockout Mice Have Increased Lipogenesis by PASK–SREBP1 Signaling and a Polarity Shift to Inflammatory Macrophages in White Adipose Tissue

**DOI:** 10.3390/cells11010004

**Published:** 2021-12-21

**Authors:** Terry D. Hinds, Zachary A. Kipp, Mei Xu, Frederique B. Yiannikouris, Andrew J. Morris, Donald F. Stec, Walter Wahli, David E. Stec

**Affiliations:** 1 Department of Pharmacology and Nutritional Sciences, University of Kentucky, Lexington, KY 40508, USA; Zachary.Kipp@uky.edu (Z.A.K.); mxu222@uky.edu (M.X.); fbyian2@uky.edu (F.B.Y.); 2 Barnstable Brown Diabetes Center, University of Kentucky, Lexington, KY 40508, USA; 3 Markey Cancer Center, University of Kentucky, Lexington, KY 40508, USA; 4 Division of Cardiovascular Medicine, College of Medicine, University of Kentucky, Lexington, KY 40508, USA; AJMorris@uams.edu; 5 Lexington Veterans Affairs Medical Center, Lexington, KY 40508, USA; 6 Small Molecule NMR Facility Core, Vanderbilt Institute of Chemical Biology, Vanderbilt University, Nashville, TN 37235, USA; donald.f.stec@vanderbilt.edu; 7 Lee Kong Chian School of Medicine, Nanyang Technological University Singapore, Clinical Sciences Building, Singapore 308232, Singapore; Walter.Wahli@unil.ch; 8 Toxalim Research Center in Food Toxicology (UMR 1331), INRAE, ENVT, INP—PURPAN, UPS, Université de Toulouse, F-31300 Toulouse, France; 9 Center for Integrative Genomics, Université de Lausanne, Le Génopode, CH-1015 Lausanne, Switzerland; 10 Department of Physiology & Biophysics, Cardiorenal and Metabolic Diseases Research Center, University of Mississippi Medical Center, Jackson, MS 39216, USA

**Keywords:** obesity, cholesterol esters, adipocyte, fatty acid synthase, FAS, SCD1, sexual dimorphism, adipogenesis, lipid signaling, inflammation

## Abstract

The nuclear receptor PPARα is associated with reducing adiposity, especially in the liver, where it transactivates genes for β-oxidation. Contrarily, the function of PPARα in extrahepatic tissues is less known. Therefore, we established the first adipose-specific PPARα knockout (*Ppara*^FatKO^) mice to determine the signaling position of PPARα in adipose tissue expansion that occurs during the development of obesity. To assess the function of PPARα in adiposity, female and male mice were placed on a high-fat diet (HFD) or normal chow for 30 weeks. Only the male *Ppara*^FatKO^ animals had significantly more adiposity in the inguinal white adipose tissue (iWAT) and brown adipose tissue (BAT) with HFD, compared to control littermates. No changes in adiposity were observed in female mice compared to control littermates. In the males, the loss of PPARα signaling in adipocytes caused significantly higher cholesterol esters, activation of the transcription factor sterol regulatory element-binding protein-1 (SREBP-1), and a shift in macrophage polarity from M2 to M1 macrophages. We found that the loss of adipocyte PPARα caused significantly higher expression of the Per-Arnt-Sim kinase (PASK), a kinase that activates SREBP-1. The hyperactivity of the PASK–SREBP-1 axis significantly increased the lipogenesis proteins fatty acid synthase (FAS) and stearoyl-Coenzyme A desaturase 1 (SCD1) and raised the expression of genes for cholesterol metabolism (*Scarb1*, *Abcg1*, and *Abca1*). The loss of adipocyte PPARα increased *Nos2* in the males, an M1 macrophage marker indicating that the population of macrophages had changed to proinflammatory. Our results demonstrate the first adipose-specific actions for PPARα in protecting against lipogenesis, inflammation, and cholesterol ester accumulation that leads to adipocyte tissue expansion in obesity.

## 1. Introduction

Despite it being over three decades since the discovery of the nuclear receptor peroxisome proliferator-activated receptor α (PPARα) [1], its role in extrahepatic tissues remains ambiguous. PPARα ligands, such as fibrates or the newly discovered endogenous ligand, bilirubin [2,3,4,5], are beneficial in reducing plasma triglyceride levels [6,7] and possibly in lowering plasma apolipoprotein B100 (ApoB100) [8,9], which both originate from the liver. While most studies have revealed the primary function of PPARα is in the liver, others have shown that the nuclear receptor also regulates body weight and adiposity [10,11,12,13]. However, most anti-adiposity mechanisms have been supported in studies using PPARα ligands that elicit whole-body responses or global PPARα knockout (KO) animals. The explicit tissue-specific mechanisms are not yet clearly understood. Mice with a global PPARα knockout (*Ppara*^−/−^) were generated over two decades ago [14], and many studies have utilized these animals for understanding signaling mechanisms. However, the results depend on the background of the *Ppara*^−/−^ mice; for example, studies of Sv/129 or C57BL/6N genetic backgrounds in these mice showed discrepancies in the development of adiposity, or that they acquire obesity in a sexually dimorphic fashion [15,16,17].

Potential inconsistencies in the former studies most likely emanated from the use of the global PPARα knockout (*Pparα*^−/−^) mice with excised exon 8 [14], which removes helix 12 of the ligand-binding domain (LBD) [18]. These animals were later found to have a smaller truncated PPARα isoform that contains the DNA-binding domain (DBD) [18], which might be hyperactive and cause some of the divergences observed in the previous studies. De Souza et al., compared the global PPARα KO mice with *Ppara* siRNA knockdown in C57/BL6 mice. They found that siRNA suppression of PPARα in mice provided a variance in responses compared to global PPARα KO animals [19]. Nevertheless, they found a high-transcriptional concordance in the magnitude and direction between *Ppara* siRNA-treated mice and global PPARα KO mice aligned using genome-wide transcriptional profiling [19]. However, these comparisons were to the WT mice treated with the PPARα ligand fenofibrate, in which the global PPARα KO mice do not contain helix 12 of the LBD, and for this reason, do not have ligand responses. Hence, the smaller truncated form without the LBD might interfere with PPARα signaling that is not visible when comparing ligand responses, which might have caused variances in the previously published works.

A consensus in past studies supports that PPARα controls hormones that regulate adiposity, especially in the liver, such as PPARα-induced fibroblast growth factor 21 (FGF21) [20], a well-established anti-obesity and anti-diabetic hepatokine hormone. Together, knowing that PPARα controls hepatokines that may also control adiposity and that global PPARα KO mice have a smaller truncated form drives the need for more studies utilizing PPARα-tissue-specific KO animals with both isoforms targeted. Recently, PPARα floxed (*Ppara*^fl/fl^) mice that excise exon 4 to remove all PPARα isoforms were generated and used to develop hepatocyte-specific PPARα KO (*Ppara*^HepKO^) animals that were shown to have hepatic steatosis on a normal-chow diet [21,22] as well as significantly worsened lipid accumulation, hepatic inflammation, and hyperlipidemia on a high-fat diet [21]. Using different PPARα floxed mice from the Gonzalez lab that excises exon 5 [23], Brocker et al. generated hepatocyte-specific PPARα KO (*Ppara*^ΔHep^) and macrophage-specific PPARα KO (*Ppara*^Δmac^) animals. Interestingly, they found that the PPARα ligand WY 14,643 controls body weight loss via the liver as the *Ppara*^Δhep^ did not lose weight with the treatment with this compound compared to mice with intact hepatic PPARα [23]. Other work by Wang et al. using the same PPARα floxed mice with exon 5 excised, showed that cardiomyocyte PPARα is essential for energy metabolism and extracellular matrix homeostasis during pressure-overload-induced cardiac remodeling [24]. Their bioenergetic analyses showed that basal and maximal oxygen consumption rates (OCR) and ATP production significantly increased in hypertrophic *Ppara*^fl/fl^ hearts, and was reduced in *Ppara*^ΔCM^ hearts. However, these studies did not use a high-fat diet challenge to determine whether the tissue-specific effects of PPARα or its ligand-induced actions were altered. For instance, Gordon et al., showed that bilirubin activated PPARα in obese animals to increase the mitochondrial number in white adipose tissue (WAT) via regulating the endogenous PPARα-interactome with coregulators that activate transcription, and this resulted in a reduction of adipocyte size and body weight [2]. 

Since PPARα has been thought to be involved in controlling adiposity [2,5,22,25,26,27,28,29], we hypothesized that the loss of adipocytic PPARα would worsen fat accumulation in adipocytes of mice fed a high-fat diet. Here, we wanted to determine the role of PPARα explicitly in adipocytes and if there is a differential effect of the loss of PPARα between females and males on normal-chow (NCD) and high-fat diets (HFD). We generated adipose-specific PPARα KO (*Ppara*^FatKO^) mice, which was the first time PPARα had only been removed from adipocytes. We present data that males had significantly more adiposity with HFD, but the female mice were protected. We found that the male *Ppara*^FatKO^ mice had elevated adipocyte cholesterol esters, a change in macrophage polarity to inflammatory, and activation of the transcription factor, sterol regulatory element-binding protein-1 (SREBP-1), which induces lipogenesis and cholesterol metabolism pathways. Our results demonstrate the first adipose-specific function for PPARα in protecting against lipogenesis, inflammation, and cholesterol-ester accumulation that leads to adipocyte hypertrophy and obesity.

## 2. Materials and Methods

### 2.1. Animals

**Figure 1:**
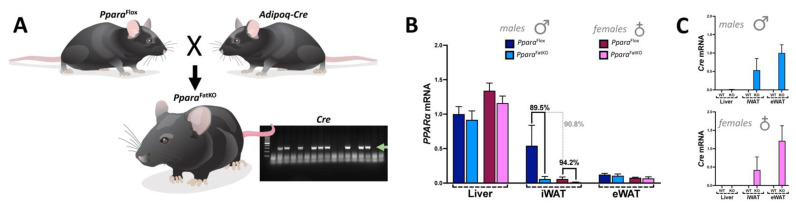
Generation of the *Ppara*^FatKO^ and *Ppara*^fl/fl^ mice. (**A**) *Ppara*^fl/fl^ and *Adipoq-Cre* mice were mated to generate the *Ppara*^FatKO^. The green arrow in the gel indicates the Cre band. Real-time PCR expression of PPARα (**B**) and Cre (**C**) in male and female *Ppara*^FatKO^ and *Ppara*^fl/fl^ mice.

*Ppara*^fl/fl^ mice were initially described in [22]. To generate adipose-specific PPARα knockout (*Ppara*^FatKO^) animals, we used adiponectin-Cre (*Adipoq*-Cre) mice on a C57BL/6J background purchased from Jackson Laboratories (stock #028020, Bar Harbor, ME). Mice were crossed with homozygous for *Ppara*^fl/fl^ and heterozygous for *Adipoq*-Cre to generate *Ppara*^FatKO^ mice and control *Ppara*^fl/fl^ littermates (Figure 1). All animals were genotyped before use to confirm either *Ppara*^FatKO^ or *Ppara*^fl/fl^ mice. Studies were performed on 8-week-old female and male mice initially housed under standard conditions with full access to standard mouse chow and water. After this time, mice were switched to a 60% high-fat diet (HFD) (diet #D12492, Research Diets, Inc., New Brunswick, NJ, USA) or normal chow (NCD) consisting of a 17% fat diet (Teklad 22/5 rodent diet, #860, Harland Laboratories, Inc., Indianapolis, IN, USA) for 30 weeks. All mice had free access to food and water ad libitum. Animals were housed in a temperature-controlled environment with a 12 h dark–light cycle. At the end of the 30-week period, mice were euthanized via overdose of isoflurane anesthesia, and tissues were immediately dissected, weighed, and frozen in liquid nitrogen. Tissue samples were stored at −80 °C until use. This study’s experimental procedures and protocols conformed to the National Institutes of Health Guide for the Care and Use of Laboratory Animals. They were approved by the Institutional Animal Care and Use Committee of the University of Mississippi Medical Center in accordance with the NIH Guide for the Care and Use of Laboratory Animals.

### 2.2. Body Composition

The changes in body composition were assessed as we have previously described [2,5,21,27,30]. In brief, the body composition was quantitated at 4-week intervals throughout the study using magnetic resonance imaging (EchoMRI-900TM, Echo Medical System, Houston, TX, USA). Conscious mice were placed in a thin-walled plastic cylinder and briefly submitted to a low-intensity electromagnetic field where fat mass, lean mass, free water, and total water were measured.

### 2.3. Analysis of Plasma Lipids and Metabolites

Nuclear magnetic resonance (NMR) spectroscopy experiments quantitating plasma lipids and metabolites were as previously described [7,21]. In brief, we used a 14.0 T Bruker magnet equipped with a Bruker AV-III console operating at 600.13 MHz. Experiment conditions included: sample temperature of 310 K, 96 k data points, 30 ppm sweep width, a recycle delay of 4 s, a mixing time of 150 ms, and 32 scans. All spectra were acquired in 3 mm NMR tubes using a Bruker 5 mm QCI cryogenically cooled NMR probe. Plasma samples were prepared and analyzed according to the Bruker In-Vitro Diagnostics research (IVDr) protocol. Sample preparation consisted of combining 50 uL of plasma with 150 uL of the buffer supplied by Bruker Biospin specifically for the IVDr protocol. For 1D ^1^H NMR, data was acquired using the 1D-NOE experiment, which filters NMR signals associated with broad line widths. Lipoprotein subclass analysis was performed using regression analysis of the NMR data, which is done automatically as part of the IVDr platform as previously described.

### 2.4. Lipidomics

Lipids were extracted using acidified organic solvents as described previously [31,32,33]. Lipid class-specific internal standards (Avanti Polar Lipids, Alabaster, AL, USA) were added at the start of the extraction. Dried lipid residues were reconstituted for analysis. The instrument system was a Shimadzu Nexera UHPLC system coupled with an AB Sciex 6500+ Q-Trap linear ion trap/triple quadrupole mass spectrometer. Lipids were separated by HILIC chromatography using a Phenomenex Luna Silica Column with a guard column of the same material and detected in multiple reaction monitoring modes using literature method adaptations. In brief, this approach takes advantage of the ability of HILIC chromatography to separate the major classes of glycero and sphingo lipids which then enables the use of time-scheduled measurements of multiple lipid species within each class. The use of weakly alkaline solvents (pH 8.0) enhances the detection of anionic lipids in negative-ionization mode. Most lipids are monitored as their precursor molecular ions, but in some cases, lipids were monitored as ammoniated adducts. The high sensitivity and speed of the instrument allow for the accurate integration of chromatographic peaks. The method used was optimized using a standard mouse-liver lipid extract to identify retention times and exclude lipid species that are present at low levels and/or not detected consistently. The final optimized method was used to analyze lipids in experimental samples, with data collected for three technical replicates of each sample. Data were analyzed using AB Sciex MultiQuant software (Framingham, MA, USA) for peak finding and integration. The raw peak areas were normalized for recovery of the appropriate internal standards. Lipids species with coefficients of variation of greater than 20% were excluded from the final report.

### 2.5. Quantitative Real-Time PCR Analysis

Total RNA was harvested from *Ppara*^fl/fl^ and *Ppara*^FatKO^ mice by lysing samples using a Qiagen Tissue Lyser LT (Qiagen, Hilden, Germany) and then extraction by a 5-Prime PerfectPure RNA Tissue Kit (Fisher Scientific Company, LLC, Waltham, MA, USA). Total RNA was read on a NanoDrop 2000 spectrophotometer (Thermo Fisher Scientific, Wilmington, DE, USA), and cDNA was synthesized using a High-Capacity cDNA Reverse Transcription Kit (Applied Biosystems, Waltham, MA, USA). PCR amplification of the cDNA was performed by quantitative real-time PCR using TrueAmp SYBR Green qPCR SuperMix (Alkali Scientific, Fort Lauderdale, FL, USA) for gene-specific primers (Table 1) and as previously described [5,20,27,30,34,35]. The thermocycling protocol consisted of 5 min at 95 °C, 40 cycles of 15 sec at 95 °C, and 30 sec at 60 °C, finished with a melting curve ranging from 60 to 95 °C to allow distinction of specific products. Normalization was performed in separate reactions with primers to 36B4.

### 2.6. Gel Electrophoresis and Western Blotting

Mouse tissues were flash-frozen in liquid nitrogen during harvesting and stored at −80 °C. For gel electrophoresis, 50–100 mg of cut tissue was then resuspended in 3 volumes of M-PER Mammalian Protein Extraction Reagent (ThermoFisher Scientific, Waltham, MA, USA, Cat no: 78501) plus 10% protease inhibitor cocktail (Sigma P2714-1BTL, Sigma Aldrich, St. Louis, MO, USA) and Halt phosphatase inhibitor cocktail (Fisher PI78420, Thermo Fisher, Waltham, MA, USA), and then incubated on ice for 30 min. The samples were lysed using a Qiagen Tissue Lyser LT (Qiagen, Hilden, Germany) and then centrifuged at 100,000× *g* at 4 °C. Protein samples were resolved by SDS polyacrylamide gel electrophoresis and electrophoretically transferred to Immobilon-FL membranes. Membranes were blocked at room temperature for 1 h in the LI-COR Intercept Blocking Buffer (LI-COR Biosciences, Lincoln, NE, USA, Cat no: 927-60001). Subsequently, the membranes were incubated overnight at 4 °C with the following antibodies: SREBP-1 (Santa Cruz Biotechnology, Dallas, TX, USA, Cat no: sc-367; 1:250 dilution in TBS), FAS (Cell Signaling, Danvers, MA, USA, Cat no: 3180s; 1:1000 dilution in TBS), SCD1 (Cell Signaling, Danvers, MA, USA, Cat no: 2794; 1:500 dilution in TBS), RPLP0 (36B4) (ThermoFisher Scientific, Waltham, MA, USA, Cat no: 11290-2-AP; 1:1000 dilution in TBS), or heat shock protein 90 (HSP90) (Santa Cruz Biotechnology, Dallas, TX, USA, Cat no: sc-13119; 1:10,000 dilution in TBS). After three washes in TBS + 0.1% Tween 20, the membrane was incubated with an infrared anti-rabbit (IRDye 800, green) or anti-mouse (IRDye 680, red) secondary antibody labeled with IRDye infrared dye (LI-COR Biosciences, Lincoln, NE, USA) (1:10,000 dilution in TBS) for 2 h at 4 °C. Immunoreactivity was visualized and quantified by infrared scanning in the Odyssey system (LI-COR Biosciences, Lincoln, NE, USA).

### 2.7. Statistical Analysis

Data were analyzed with Prism 9 (GraphPad Software, San Diego, CA, USA) using analysis of variance combined with Tukey’s post-hoc test to compare pairs of group means or unpaired *t*-tests. Results are expressed as mean ± SEM. Additionally, a one-way ANOVA with a least significant difference post-hoc test was used to compare mean values between multiple groups, and a two-tailed and a two-way ANOVA was utilized in multiple comparisons, followed by the Bonferroni post-hoc analysis to identify interactions. *p* values of 0.05 or smaller were considered statistically significant.

## 3. Results

### 3.1. Adiposity in Adipocyte-Specific PPARα Knockout and Control Littermate Mice on High-Fat and Regular-Chow Diets

**Figure 2:**
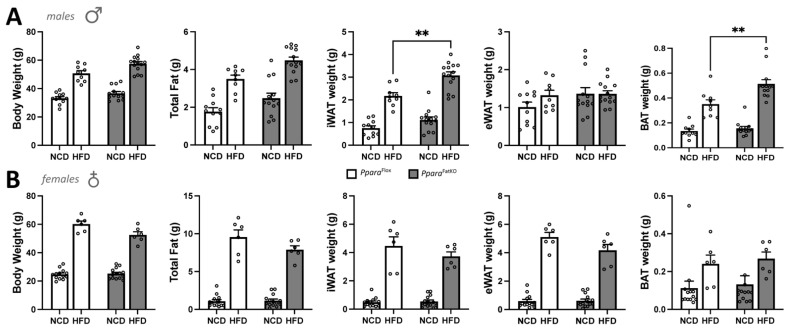
Body and fat-pad weights of *Ppara*^FatKO^ and *Ppara*^fl/fl^ mice fed high-fat or normal-chow diets. Male (**A**) and female (**B**) *Ppara*^FatKO^ and *Ppara*^fl/fl^ mice were placed on high-fat (HFD) or normal-chow (NCD) diets for 30 weeks and body weights, total fat, inguinal white adipose tissue (iWAT), epididymal white adipose tissue (eWAT), and brown adipose tissue (BAT) were measured at the end of the study. ** *p* < 0.01, two-way ANOVA (vs. *Ppara*^fl/fl^ HFD) (± S.E.M.; male *n* = 11 NCD and *n* = 9 HFD, *Ppara*^fl/fl^; female *n* = 13 NCD and *n* = 6 HFD, *Ppara*^fl/fl^; male *n* = 13 NCD and *n* = 14 HFD, *Ppara*^HepKO^; female *n* = 14 NCD and *n* = 6 HFD, *Ppara*^HepKO^).

While many studies have supported a role for PPARα in reducing body weight and possibly adiposity, the definitive function of PPARα in adipose tissue has not been resolved. Therefore, we wanted to determine the function of PPARα explicitly in adipocytes and whether an adipocyte-specific loss of PPARα (*Ppara*^FatKO^) causes metabolic disturbances or adiposity. Here, we generated mice using *Ppara*^fl/fl^ described in [21,22] crossed with mice containing an adiponectin-Cre (*Adipoq*-Cre) to establish an adipocyte-specific KO (Figure 1), as we have previously described [36]. Analysis of the tissue distribution of PPARα in *Ppara*^fl/fl^ and *Ppara*^FatKO^ indicated that expression was reduced 89.5% in males and 94.5% in females in inguinal white adipose tissue (iWAT) (also referred to as subcutaneous fat) and to a lesser extent in brown adipose tissue (BAT) (data not shown). Very low PPARα expression was observed in epididymal white adipose tissue (eWAT) for males and females; this same observation has been reported previously [37]. To determine whether the absence of PPARα would impact adiposity and fat-pad size, we fed the *Ppara*^FatKO^ and littermate control (*Ppara*^fl/fl^) mice a normal-chow diet (NCD) or high-fat diet (HFD) for 30 weeks. We found that the male *Ppara*^FatKO^ mice compared to *Ppara*^fl/fl^ on an HFD had significantly higher iWAT (two-way ANOVA, *p* = 0.0091) and BAT (two-way ANOVA, *p* = 0.0031), but no significant differences in body weight (two-way ANOVA, *p* = 0.0840), total fat (two-way ANOVA, *p* = 0.0982), or eWAT (two-way ANOVA, *p* = 0.9991) between the groups (Figure 2A). Also, there were no differences between heart or liver weights for NCD or HFD for the males (data not shown). The female *Ppara*^FatKO^ compared to the female *Ppara*^fl/fl^ mice on NCD or HFD had no significant differences in body weights, fat mass, iWAT, eWAT, BAT (Figure 2B), heart, or liver weights (data not shown). It should be noted that there was a trend for these measurements to be higher for males in the HFD group for body weight and total fat between the *Ppara*^FatKO^ and *Ppara*^fl/fl^ mice but lower for females for all HFD groups. However, these observations were not significant. The eWAT was larger by HFD feeding in the females for both genotypes, but no change was observed in the males. This may have been due to the intrinsically low levels of PPARα in eWAT [37]. Mice on an HFD [38] or those that are genetically ob/ob obese [20,38] have been demonstrated to have reduced PPARα expression in adipose and liver tissues. When comparing the HFD to NCD in the *Ppara*^fl/fl^ animals, the PPARα expression in iWAT was reduced by 94.65% in males and 88.46% in females by the HFD (data not shown). In comparing sex differences for PPARα levels, the expression of PPARα was 90.8% lower in the females compared to the males for the iWAT of the *Ppara*^fl/fl^ mice on normal chow (Figure 1). Therefore, we deduce that there is a sexual dimorphism for PPARα in adipose tissue. Male mice with an adipocyte-specific loss of PPARα (*Ppara*^FatKO^) have increased adiposity in fat depots iWAT and BAT, but this was not observed in females. The latter is presumably related to estrogen. Previous work has shown that estrogen suppresses PPARα transcriptional activity [11,39,40,41] and controls adipocyte size [42]. This indicate that estrogen in females offers a protective factor in reducing adiposity independent of PPARα adiposity-reducing mechanisms.

### 3.2. Analysis of Serum Metabolites and Cholesterol in Adipocyte-Specific PPARα Knockout and Control Littermate Mice

**Figure 3:**
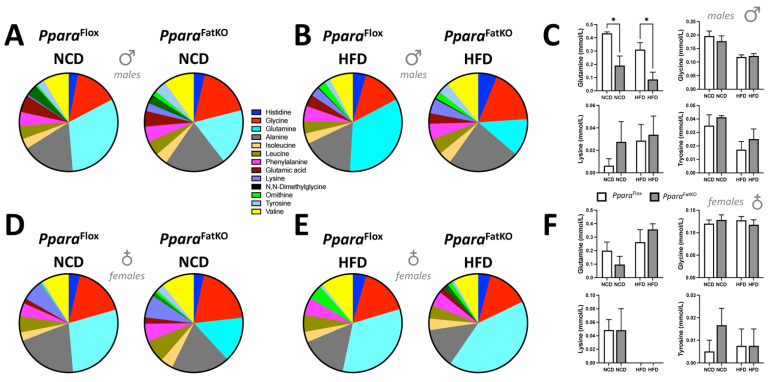
Plasma amino acids in *Ppara*^FatKO^ and *Ppara*^fl/fl^ mice fed high-fat or normal-chow diets. Pie and bar graphs of plasma amino acids in male (**A**–**C**) and female (**D**–**F**) *Ppara*^FatKO^ and *Ppara*^fl/fl^ mice on high-fat (HFD) or normal-chow (NCD) diets. * *p* < 0.05 (vs. *Ppara*^fl/fl^ NCD, HFD) (male *n* = 8 NCD and *n* = 7 HFD, *Ppara*^fl/fl^; female *n* = 6 NCD and *n* = 4 HFD, *Ppara*^fl/fl^; male *n* = 8 NCD and *n* = 8 HFD, *Ppara*^HepKO^; female *n* = 6 NCD and *n* = 4 HFD, *Ppara*^HepKO^).

Since adipose serves as a large depot for fat and other metabolites and hormones, we wanted to determine if the experimental animals had changes in blood-metabolite or cholesterol levels that PPARα might mediate in adipocytes. The plasma metabolites such as amino, carboxylic, and keto acids were analyzed using the nuclear magnetic resonance (NMR) spectroscopy approach that we have previously described [7,21]. Quantitation of plasma amino acids showed that the only significant differences between any groups, as measured by two-way ANOVA, in NCD, HFD, and for sex, were for plasma glutamine, which was significantly lower in the male *Ppara*^FatKO^ compared to littermate control mice on an NCD or HFD (Figure 3A–F). Please note that the comparisons were made for NCD WT vs. NCD KO and HFD WT vs. HFD KO, and this pattern will be used in the figures herein. The *Ppara*^FatKO^ and *Ppara*^fl/fl^ female mice had no significant differences between these groups for the measurement of plasma glutamine levels. Also, the levels were not significantly different between the HFD and NCD for the *Ppara*^FatKO^ group in the males, indicating that this metabolite is most likely not a primary player causing adipocyte size changes.

**Figure 4:**
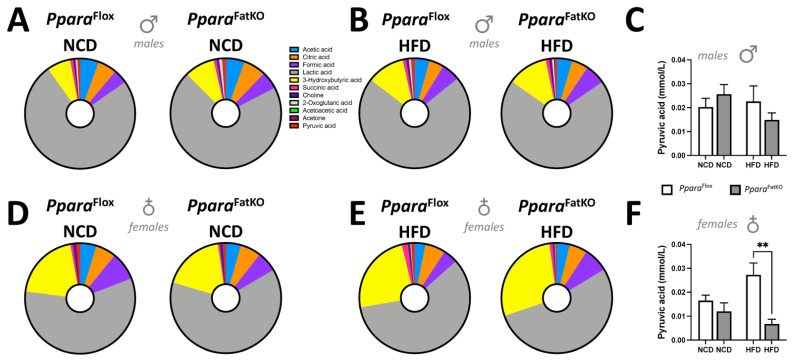
Plasma metabolites in *Ppara*^FatKO^ and *Ppara*^fl/fl^ mice fed high-fat or normal-chow diets. Pie and bar graphs of plasma amino acids in male (**A**–**C**) and female (**D**–**F**) *Ppara*^FatKO^ and *Ppara*^fl/fl^ mice on high-fat (HFD) or normal-chow (NCD) diets. **, *p* < 0.01 (vs. *Ppara*^fl/fl^ HFD) (male *n* = 8 NCD and *n* = 7 HFD, *Ppara*^fl/fl^; female *n* = 6 NCD and *n* = 4 HFD, *Ppara*^fl/fl^; male *n* = 8 NCD and *n* = 8 HFD, *Ppara*^HepKO^; female *n* = 6 NCD and *n* = 4 HFD, *Ppara*^HepKO^).

PPARα controls β-oxidation to produce the major fatty acid metabolite and ketone, β-hydroxybutyrate (BHOB), which is released from the liver into the blood [43]. There were no significant differences for the *Ppara*^FatKO^ and *Ppara*^fl/fl^ mice on HFD or NCD for plasma levels of BHOB (measured as 3-hydroxybutyric acid, the yellow section on the pie graph) (Figure 4A–F). This result could be anticipated as BHOB comes primarily from the liver [44]. The results in females show that pyruvic acid was significantly lower in the *Ppara*^FatKO^ HFD-fed group compared to littermate control mice fed the same diet (Figure 4F). These results might be related to PPARα regulation of pyruvate dehydrogenase activity in adipose tissue of females, as it is known to regulate this activity [45]. The metabolite data essentially show no differences among the groups or genotypes. 

**Figure 5:**
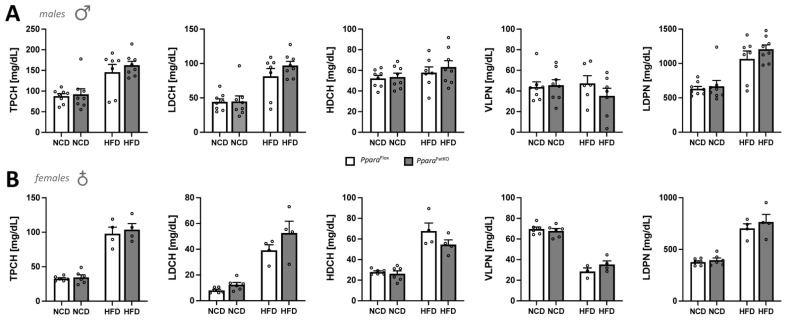
Plasma total cholesterol, VLDL, and LDL particle numbers in *Ppara*^fl/fl^ and *Ppara*^FatKO^ mice. Total plasma cholesterol (TPCH), LDL cholesterol (LDCH), HDL cholesterol (HDCH), VLDL particle number (VLPN), and LDL particle number (LDPN) as measured by NMR spectroscopy in plasma of male (**A**) and female (**B**) *Ppara*^FatKO^ and *Ppara*^fl/fl^ mice on high-fat (HFD) or normal-chow (NCD) diets. (male *n* = 8 NCD and *n* = 7 HFD, *Ppara*^fl/fl^; female *n* = 6 NCD and *n* = 4 HFD, *Ppara*^fl/fl^; male *n* = 8 NCD and *n* = 8 HFD, *Ppara*^HepKO^; female *n* = 6 NCD and *n* = 4 HFD, *Ppara*^HepKO^).

Since the adipose tissue is a large depot for free cholesterol [46], and PPARα is a drug target for treating hyperlipidemia and is well known to regulate lipid levels, we next wanted to determine whether adipocytic PPARα affects plasma cholesterol and lipoprotein levels. The results show no significant differences between the *Ppara*^FatKO^ and *Ppara*^fl/fl^ groups for male or female mice on NCD or HFD for total cholesterol (TPCH), LDL (LDCH), HDL (HDCH), VLDL (VLPN), or LDL particle number (LDPN) (Figure 5A,B). However, it should be noted that most of these lipids were significantly changed between the diets (NCD vs. HFD) for each individual genotype. Overall, there were no significant differences when comparing littermate control and adipocyte-specific PPARα knockout mice for plasma cholesterol and lipoprotein levels. 

### 3.3. Lipidomics and Signaling in Adipocyte-Specific PPARα Knockout and Littermate Control Mice

**Figure 6:**
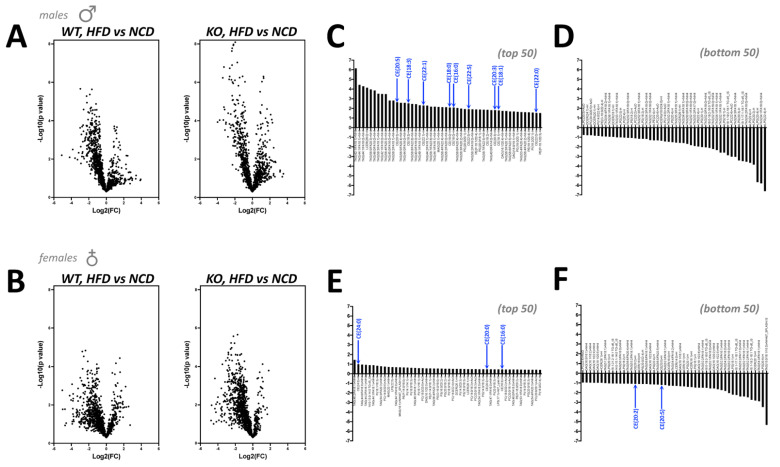
Lipidomics of iWAT in *Ppara*^fl/fl^ and *Ppara*^FatKO^ mice. Lipidomics of iWAT using liquid chromatography–mass spectrophotometry (LC mass spec) represented as volcano-plot analysis in male (**A**) and female (**B**) *Ppara*^FatKO^ and *Ppara*^fl/fl^ mice on high-fat (HFD) or normal-chow (NCD) diets. Organization of the top 50 and bottom 50 most-changed lipids in male (**C**,**D**) and female (**E**,**F**) *Ppara*^FatKO^ and *Ppara*^fl/fl^ mice. The blue arrows identify cholesterol esters. (male *n* = 7 NCD and *n* = 8 HFD, *Ppara*^fl/fl^; female *n* = 6 NCD and *n* = 4 HFD, *Ppara*^fl/fl^; male *n* = 7 NCD and *n* = 8 HFD, *Ppara*^HepKO^; female *n* = 6 NCD and *n* = 4 HFD, *Ppara*^HepKO^).

The above results indicate that adipocyte PPARα does not regulate plasma levels of amino acids, metabolites, or cholesterol, or lipoprotein metabolism. Therefore, we wanted to specifically investigate the adipose tissue responses in iWAT as this was significantly different for the male *Ppara*^FatKO^ on HFD compared to littermate controls on the same diet. Using iWAT from all groups of mice, we performed lipidomics using liquid chromatography–mass spectrophotometry (LC mass spec). Volcano-plot analysis of the lipidomics data showed that male *Ppara*^FatKO^ had more significant responses, as related to the higher values on the y-axis for the HFD vs. NCD when compared to *Ppara*^fl/fl^ littermates (Figure 6A). The female mice did not show similar significant differences when comparing the y- axis for the *Ppara*^FatKO^ to the *Ppara*^fl/fl^ (Figure 6B). Next, we wanted to compare the volcano plots in the *Ppara*^FatKO^ to the *Ppara*^fl/fl^ for males and females. To determine the lipid species that were most changed, we quantitated the differences between the diet groups and *Ppara*^FatKO^ versus *Ppara*^fl/fl^ mice (KO(HFD/NCD)–WT(HFD/NCD)). We also wanted to determine if there were any clusters of specific lipids visible that might indicate signaling pathways that control their generation. This analysis showed that the *Ppara*^FatKO^ mice had several triacylglycerides (TAGs) that were higher in the males (Figure 6C,D). The specific TAGs that were raised in the male *Ppara*^FatKO^ mice were TAG 42:1/FA18:1, TAG 44:1/FA18:1, TAG 44:1/FA16:0, TAG 56:5/FA20:3; TAG 46:0/FA18:0, and TAG 42:1/FA16:0. The female *Ppara*^FatKO^ mice did not show similar patterns, and the changes were overall very low (Figure 6E,F). The male mice had a cluster showing that cholesterol esters were elevated as indicated by blue arrows (Figure 6C), but this was not observed at the same level in females (Figure 6E). 

**Figure 7:**
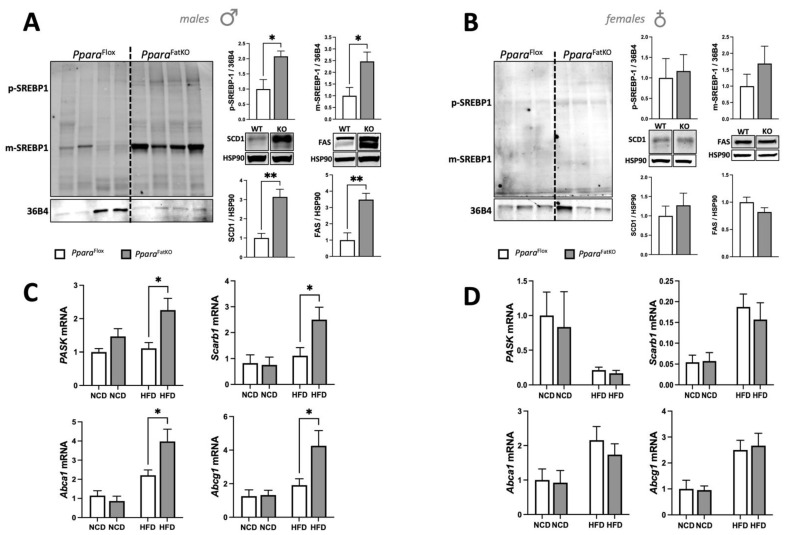
PASK–SREBP-1 signaling in iWAT of *Ppara*^fl/fl^ and *Ppara*^FatKO^ mice. Western blotting and densitometry of SREBP-1 and 36B4 as the control, and stearoyl-coenzyme A desaturase 1 (SCD1) and fatty acid synthase (FAS) and heat shock protein-90 (HSP90) as control in *Ppara*^fl/fl^ and *Ppara*^FatKO^ on HFD in (**A**) male and (**B**) female mice. * *p* < 0.05, ** *p* < 0.01, Student-*t* test (versus *Ppara*^fl/fl^ HFD) (± S.E.M.; male and female, *n* = 6 for FAS and SCD1 male; *n* = 4 for SREBP1 for male and female). Real-time mRNA expression for *Pask*, *Scarb1*, *Abca1*, and *Abcg1* in *Ppara*^fl/fl^ and *Ppara*^FatKO^ on NCD or HFD in (**C**) male and (**D**) female mice. * *p* < 0.05, two-way ANOVA (versus HFD *Ppara*^fl/fl^) (male *n* = 7 NCD and *n* = 7 HFD, *Ppara*^fl/fl^; male *n* = 8 NCD and *n* = 8 HFD, *Ppara*^HepKO^).

These results indicate that intracellular signaling in iWAT could be changed specifically in males, but not females, as this causes higher lipids and cholesterol esters. Therefore, we measured the master regulator of an intracellular cholesterol signaling molecule, SREBP-1, and whether the mature form, which is the only form that can function as a transcription factor [47], was more present in the *Ppara*^FatKO^ male mice on HFD. We found that SREBP-1 mRNA was unchanged (data not shown), but the mature form of the SREBP-1 protein was significantly higher in the *Ppara*^FatKO^ male mice on HFD (Figure 7A). The female *Ppara*^FatKO^ mice on HFD did not have a high level of the mature SREBP1 form (Figure 7B). The SREBP-1 transcription factor has been shown to cause lipid accumulation by regulating the expression of fatty acid synthase (FAS) and stearoyl-coenzyme A desaturase 1 (SCD1) [48]. Immunoblotting of iWAT from the *Ppara*^FatKO^ and *Ppara*^fl/fl^ male mice on HFD showed that SCD1 and FAS were significantly higher (*p* < 0.01) in the *Ppara*^FatKO^ male mice on HFD compared to the *Ppara*^fl/fl^ mice on the same diet (Figure 7A). The female mice on HFD showed no significant differences for SCD1 or FAS expression (Figure 7B).

A recent study has shown that the Per-Arnt-Sim kinase (PASK) controls the mature form of SREBP1, which impacts the overall accumulation of lipids [49]. PASK mRNA levels were significantly higher in the *Ppara*^FatKO^ male mice on HFD than in the *Ppara*^fl/fl^ mice on the same diet (Figure 7C). The female mice fed NCD or HFD had no significant differences in PASK expression between the groups (Figure 7D). These results indicate that the PASK–SREBP-1 axis was elevated in the *Ppara*^FatKO^ male mice on HFD compared to the *Ppara*^fl/fl^ mice on the same diet. This notion is further supported by the SREBP-1 target genes *Scarb1, Abca1*, and *Abcg1* being significantly higher in the males on HFD and no change with NCD (Figure 7C). These genes are known regulators of intracellular free cholesterol and cholesterol esters levels. There were no significant differences for these genes in female mice between the *Ppara*^FatKO^ and the *Ppara*^fl/fl^ mice (Figure 7D). 

**Figure 8:**
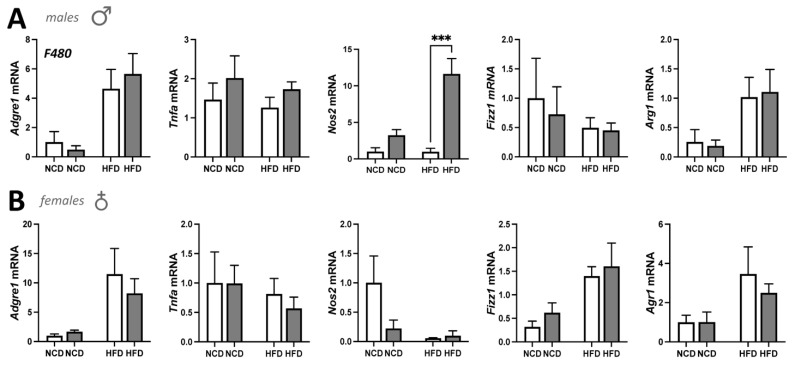
Macrophage marker expression in iWAT of *Ppara*^fl/fl^ and *Ppara*^FatKO^ mice. Real-time mRNA expression for *Adgre1* (F480), *Tnfa*, *Nos2*, *Fizz1,* and *Arg1* in *Ppara*^fl/fl^ and *Ppara*^FatKO^ on NCD or HFD in (**A**) male and (**B**) female mice. *** *p* < 0.001, two-way ANOVA (versus HFD *Ppara*^fl/fl^) (male *n* = 7 NCD and *n* = 7 HFD, *Ppara*^fl/fl^; male *n* = 8 NCD and *n* = 8 HFD, *Ppara*^HepKO^).

We have previously shown that mice with a hepatocyte-specific deletion of PPARα had worsened inflammation in the liver on an HFD [21]. We also demonstrated that glucocorticoid receptor beta (GRβ) suppresses PPARα in the liver to induce inflammation and adiposity [34]. Because of these previously published works, we wanted to determine whether the loss of PPARα in adipose tissue would have a similar effect. Adipose tissue is a mix of adipocytes and immune cells, mostly macrophages [50]. We, therefore, analyzed the macrophage marker F480 (*Adgre1* gene) and found that there were no statistically significant differences between the four groups for males or females (Figure 8). We further analyzed iWAT for macrophage subpopulations to determine whether the population polarity had possibly switched from M2 anti-inflammatory to M1 pro-inflammatory cells as we have previously described [34,36]. The results in the male mice show that pro-inflammatory marker TNFα (*Tnfa*) was slightly elevated but not statistically significant. However, the pro-inflammatory marker iNOS (*Nos2*) was significantly (*p* < 0.001) higher in *Ppara*^FatKO^ males compared to *Ppara*^fl/fl^, which induced a change in the macrophage polarity, shifting the population to M1 pro-inflammatory. These findings were not observed in females. The iNOS levels in the NCD group were also raised but not significantly (*p* = 0.5613). The M2 anti-inflammatory markers *Arg1* and *Fizz1* (also referred to as *Retnla*) had no differences between any of the four groups. The female mice appeared to have no differences in M1 or M2 markers or change in macrophage polarity. 

**Figure 9:**
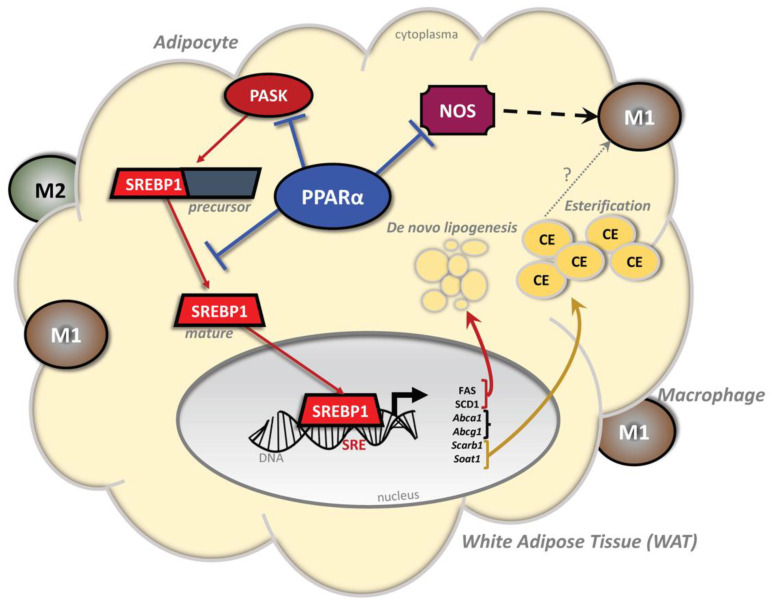
Schematic diagram of the proposed pathway in male mice by which PPARα inhibition of the PASK–SREBP-1 axis reduces lipogenesis, inflammation, and cholesterol esterification. In adipocytes, PPARα inhibits PASK expression and activation of SREBP-1. Typically, the SREBP-1 transcription factor, when activated, is truncated to the mature form, which can bind to sterol response elements (SREs) in gene promoters to control the expression of genes for lipogenesis (stearoyl-coenzyme A desaturase 1 (SCD1) and fatty acid synthase (FAS)) and cholesterol metabolism (*Abca1*, *Abcg1*, and *Scarb1*). The loss of PPARα in adipose tissue causes a significant increase in iNOS, which mediates the switch of macrophage polarity from anti-inflammatory M2 to pro-inflammatory M1. Abbreviations: PPARα, peroxisome proliferator-activated receptor-alpha; SREBP-1, sterol regulatory element-binding protein-1; PASK, Per-Arnt-Sim Kinase; CE, cholesterol ester; SRE, sterol response element.

Altogether, these data indicate that the PASK–SREBP-1 axis is hyperactive with the adipocytic loss of PPARα in males but not females, and similarly, the loss of PPARα induces a change in macrophage polarity to pro-inflammatory. A high-fat diet activates the SREBP-1 pathway in the *Ppara*^FatKO^ male mice, which induces genes that regulate lipogenesis and cholesterol metabolism (Figure 9).

## 4. Discussion

A considerable amount of published work has demonstrated the beneficial actions of PPARα ligands in reducing plasma triglycerides and fat accumulation in the liver. Until the present study, the explicit role of PPARα in adipocytes had not been fully explicated. Our findings show that PPARα in adipose tissue regulates adiposity and inflammation in males, similarly to its role in the liver [21]. However, in the study, the females were peculiar and did not present any observed differences with PPARα removed from their adipocytes. This most likely occurred from the protective actions of estrogen on adipocyte hypertrophy [42]. Yoon et al. showed that fenofibrate treatments in female mice did not reduce adiposity as observed in the males; however, ovariectomized females responded to fenofibrate and had reduced fat mass [11]. In comparing our study’s observation to this investigation, together, the results indicate that PPARα is dispensable in adipocytes for females, at least for those who have not undergone menopause. However, whether fenofibrate has improved actions in female patients with adiposity after menopause remains to be answered.

Investigations have shown that fenofibrate inhibits adipocyte hypertrophy and insulin resistance by activating adipose PPARα in HFD-induced obese male mice [10,13]. Our results in males suggest that iWAT and BAT adipose depots express a responsive PPARα, which is why they were significantly changed. However, no differences in eWAT of the males were detected. Others have shown that eWAT has almost no expression of PPARα [37]. Therefore, the changes in iWAT and BAT and no response in eWAT in the males could be anticipated. Each fat depot has specific actions [51], and sexual dimorphism has been shown to have differential responses in iWAT and eWAT [52]. As per metabolic disorders, the iWAT, more commonly referred to in humans as subcutaneous fat, is typically the most prominent fat depot [51,53]. Expansion of the WAT pads is considered to worsen obesity-related comorbidities by secreting inflammatory cytokines [54,55,56]. The primary function of iWAT is to store triglycerides and free cholesterol [57,58]. The finding that cholesterol esters were raised in the *Ppara*^FatKO^ male mice on HFD compared to the *Ppara*^fl/fl^ on the same diet is new and is an unexpected role for PPARα. Fish oil, which activates PPARα and PPARγ, has been shown to significantly reduce cholesterol esters (0.2+/− fish oil versus 1.2+/− olive oil) in WAT of *Ldlr*^−/−^ mice [59]. Interestingly, PPARγ increases adipocyte cholesterol levels [60]. Inversely, Chinetti et al. showed that activation of PPARα with different ligands reduced cholesterol esters in macrophages, reducing foam-cell formation that causes atherosclerosis [61]. The function of PPARα and PPARγ in adiposity has been considered opposing, as PPARγ regulates adipogenesis and lipogenesis [35,62,63], while PPARα reduces fat accumulation [5,7,27,30]. 

The master regulator of cholesterol, SREBP-1, regulates the balance of intracellular fat levels and lipogenesis [64]. SREBP-1 is increased with adiposity [64] and is a factor that drives gene transcription of lipogenic genes such as FAS and SCD1 [48,64,65]. We found that the *Ppara*^FatKO^ male mice on an HFD had more of the mature active form of SREBP-1, leading to higher expression of target genes such as SCD1 and FAS (Figure 9). The higher levels of FAS and SCD1 likely caused higher TAGs in the iWAT, as these specific lipids are known to be produced by SCD1 [66] and FAS [67]. The elevated expression of FAS and SCD1 was most likely related to PPARα regulation of SREBP-1 and PASK, and a loss of PPARα caused their activation. In our previously published work, we have shown that bilirubin-induced PPARα activity suppresses SREBP-1 in the liver of obese mice [27] and that FAS and SCD1 were also reduced, which lowered adiposity [5,7,27,30]. The finding that PASK was significantly higher in the *Ppara*^FatKO^ male mice on an HFD compared to littermate controls on the same diet suggests that PPARα might control PASK levels to inhibit the PASK–SREBP-1 axis from reducing adiposity. 

The PASK KO mice had significantly higher PPARα and heme oxygenase-1 (HO-1) expression in the liver and increased mitochondrial function [68]. We have shown that activation of HO-1 in obese mice enhanced PPARα and its target genes and reduced hepatic lipid accumulation [20]. We have also shown that knockout of BVRA increased lipid accumulation [26,30,36,69,70], which correlated with reduced PPARα levels and target genes [30,36]. The HO-1–BVRA–bilirubin–PPARα axis most likely antagonizes the PASK–SREBP-1 signaling. Dongil et al. showed PASK deficiency activated antioxidant mechanisms in the liver and increased HO-1 and PPARα levels [71]. They also demonstrated that the PASK knockout mice are resistant to dietary-induced obesity, are protected from glucose intolerance on an HFD, and have significantly lower plasma triglyceride levels [71]. This was supported by Pérez-García et al., who showed PASK-deficient mice are protected from metabolic complications [72,73]. They also found that PPARα was higher in the livers of the PASK knockout mice and that FAS and SCD1 were significantly lower [72,73]. Hao et al. showed that the PASK KO animals had reduced body weight on an HFD and significantly fewer liver triglycerides [74]. For this reason, Zhang et al. identified PASK as an emerging regulator of glucose and lipid metabolism [75]. These studies indicate that there might be a reciprocal relationship between PPARα and PASK, and based on our results, it is possible that PPARα directly represses PASK expression. However, more studies are needed to determine the molecular mechanism by which PPARα represses PASK.

The primary function of PPARα has been studied in the liver. Our study, here, on PPARα in adipocytes showed that BOHB was not changed between the adipocyte-specific PPARα knockout and floxed mice of both sexes. These results could be anticipated because this metabolite almost exclusively comes from the liver during fat utilization and burning [43]. BOHB is produced by ketogenesis, which is a series of reactions that lead to the formation of ketone bodies. PPARα via a transcriptional network mediates ketogenesis through ACOX1, AMP-activated protein kinase (AMPK), PPARγ coactivator 1α (PGC-1α), mammalian target of rapamycin (mTOR), and fibroblast growth factor 21 (FGF21) [76]. We have shown that bilirubin-induced PPARα activity in obese mice increased ACOX1 and BOHB inducing β-oxidation and utilization of hepatic lipids, which improved liver function as measured by a reduction in aspartate aminotransferase (AST) level, a hepatic dysfunction marker, and suppression of liver inflammation [7]. Hence, bilirubin via activation of PPARα has protective actions and reduces adiposity [77,78,79,80,81,82,83].

While total macrophage population marker F480 (*Adgre1*) was not different between the groups, iNOS (*Nos2*) proinflammatory marker was significantly higher. Adipose tissue expansion in obesity causes a phenotypic switch in macrophage polarization, and iNOS is a well-established marker for such polarity shift [21,34,84]. Becerril et al. demonstrated that *Nos2* knockout mice do not develop adipose tissue inflammation in leptin-deficient ob/ob mice [85]. We showed that hepatocyte-specific PPARα KO animals on HFD had significantly higher hepatic iNOS and worsened inflammation with a polarity shift in macrophage markers to proinflammatory [21]. Marino et al. also showed that adenoviral overexpression of the inflammatory factor GRβ reduced PPARα in the liver of mice on normal chow [34]. GRβ increased iNOS inducing a switch in the immune cell population from anti-inflammatory macrophages to proinflammatory, causing significantly more liver-fat accumulation [34]. GRβ is the inhibitory GR isotype that suppresses cortisol responsiveness [56,86,87] leading to inflammation [34,56,87,88,89,90,91] and adiposity [34,56]. Hence, the function of PPARα is to control pathways that increase fat burning, lessening adiposity and inflammation [2,4,5,27,28,29].

## 5. Conclusions

In conclusion, our study and work from others indicate that PPARα responses are sexually dimorphic and that estrogen may either suppress PPARα expression or inhibit signaling. Estrogen has anti-adiposity mechanisms that protect females from gaining as much weight as males on a high-fat diet [42]. The use of PPARα ligands in obese male mice reduces body weight [2,5,7], which might not be beneficial for females until after menopause. However, more studies are needed to understand these effects better in females. We also conclude that adipocytic PPARα does not regulate plasma levels of cholesterol such as LDL or HDL, but does regulate the intracellular level of cholesterol esters, as was previously shown in macrophages [61]. The importance of PPARα regulation of cholesterol esters in adipocytes is unclear, and more studies are needed to elucidate the function. Our investigation here reflects the first explicit function of PPARα in adipose tissue, which was shown to be fundamental in regulating the PASK–SREBP-1 axis to control lipogenesis and FAS and SCD1 expression (Figure 9). The role of PPARα in the regulation of macrophage polarity and inflammation could be mediated via PASK or vice versa. Future investigations could reveal whether PPARα directly downregulates PASK, which appears to be an essential kinase that might induce metabolic dysfunction. 

## Figures and Tables

**Table 1:** Primer sequences used in this study.

Gene	Forward Primer	Reverse Primer
*36B4*	CACTCTCGCTTTCTGGAGGG	ACGCGCTTGTACCCATTGAT
*Cre*	GAACCTGATGGACATGTTCAGG	AGTGCGTTCGAACGCTAGAGCCTGT
*Ppara*	AGAAGTTGCAGGAGGGGATT	TTGAAGGAGCTTTGGGAAGA
*Pask*	GAATCCGACTGAGACTTGCG	TAACTAACACTCGCCGCCAC
*Scarb1*	CCCCAGGTTCTTCACTACGC	TCCTTATCCTGGGAGCCCTT
*Abca1*	GGCAATGAGTGTGCCAGAGTTA	TAGTCACATGTGGCACCGTTTT
*Abcg1*	TCCCCACCTGTAAGTAATTGCA	TCGGACCCTTATCATTCTCTACAGA
*Adgre1*	GCCCAGGAGTGGAATGTCAA	GCAGACTGAGTTAGGACCACA
*Nos2*	CCTTGGTGAAGGGACTGAGC	TCCGTGGAGTGAACAAGACC
*Fizz1*	GGGATGACTGCTACTGGGTG	TCAACGAGTAAGCACAGGCA
*Arg1*	AAGAGTCAGTGTGGTGCTGG	TGGTTGTCAGGGGAGTGTTG
*Tnfa*	GACTCAAATGGGCTTTCCGA	TCCAGCCTCATTCTGAGACAGAG

## Data Availability

The data presented in this study are available on request from the corresponding authors.
